# A Novel 8 Ps Framework for Addressing Complexities in Sexual Health Histories for US Healthcare Providers

**DOI:** 10.1111/psrh.70037

**Published:** 2025-10-24

**Authors:** Brenice Duroseau, Ragan Johnson

**Affiliations:** 1 Johns Hopkins University School of Nursing, Baltimore, Maryland, USA; 2 Duke University School of Nursing, Durham, North Carolina, USA

**Keywords:** HIV, patient–provider communication, sexual health history, STIs

## Abstract

**Context:**

Healthcare providers play a vital role in promoting comprehensive, sex-positive sexual health care. In the United States (US), patient–provider communication frameworks for sexual health predominantly rely on collecting the sexual health history. The most notable framework, the US Centers for Disease Control and Prevention’s *5 Ps framework*, emphasizes how an individual’s current and past behaviors can predict a client’s susceptibility to poor sexual health outcomes. However, this behavior centric approach has the capacity to introduce bias, stigma, and shame, potentially hindering effective communication and preventative care. The National Coalition for Sexual Health’s *6 Ps framework* introduced “Plus” to include sexual satisfaction as an integral part of sexual health communication.

**Methods:**

Building on these existing frameworks, we developed an expanded model designed to reorient providers toward a paradigm that fosters more inclusive and affirming sexual health discussions, improves patient–provider communication and connection, and acknowledges the broader social and structural determinants that shape sexual well-being and vulnerability to human immunodeficiency virus (HIV) and other sexually transmitted infections (STIs).

**Results:**

We propose an expanded 8 Ps sexual health history that introduces “Proximity,” which refers to the influence of one’s living and sexual environment, and “Perspectives,” highlighting how personal beliefs fundamentally shape behaviors.

**Conclusion:**

Fortifying the sexual health discussions between providers and patients with non-behavioral context, such as geographical determinants of health and understanding of sexual health in general, that increase vulnerability to HIV and other STIs can begin to address the limitations in the prior frameworks.

## 1 | Introduction

In the United States (US), sexually transmitted infections (STIs) and human immunodeficiency virus (HIV) remain a threat to public health [1, 2], with persistent and disproportionate prevalence and incidence rates among racially and socioeconomically marginalized populations (i.e., Black women) despite decades of prevention efforts and public health investment. For example, Black women account for over 50% of new diagnoses of HIV among all women, despite only being 13% of the population [3].

Using sexual health histories (SHHs) is fundamental to the provision of effective sex-positive sexual health care. SHHs not only inform clinical decision making but also build trust through patient-provider communication and support prevention strategies (e.g., consistent condom use, regular STI and HIV testing, and pre-exposure prophylaxis [PrEP]/post-exposure prophylaxis [PEP]) for individuals most susceptible to STIs and HIV [4]. PrEP, available in oral and injectable forms, is up to 99% effective at preventing HIV, and PEP, an oral biomedical intervention that is over 80% effective when taken as prescribed within 72 h, remains [5] under prescribed and thus underutilized in Black communities [6], despite the significant potential benefits. Equally important is the concept of “Undetectable = Untransmittable” (U=U), which establishes that individuals living with HIV who achieve and maintain viral suppression cannot sexually transmit the virus to others [7–10]. U=U is a cornerstone of HIV prevention, advancing both clinical management and stigma reduction.

Healthcare providers have traditionally relied on the US Centers for Disease Control and Prevention’s (CDC) 5 Ps framework [11] (Partners, Practices, Protection from STIs, Past history of STIs, and Pregnancy plans) to guide sexual health discussions with patients. The 6 Ps framework is an extension of this model, introduced by the National Coalition for Sexual Health [12] to capture a more nuanced picture of sexual health. The 6th P, Plus, explores sexual satisfaction, function, and support for one’s individual sexuality. These models have shown success in increasing screening of chlamydia and other STIs [13–15]. However, given the complexities of sexual health, particularly within populations disproportionately impacted by STIs and HIV, existing frameworks fall short of comprehensively addressing major contributors to STIs and HIV.

Existing 5 Ps and 6 Ps models have diverse and significant limitations: (1) They often focus too narrowly on behaviors, neglecting the broader contexts within which these behaviors occur; (2) they lack the cultural and social sensitivity required to address the unique challenges faced by various demographic groups, especially those most vulnerable to HIV; and (3) they do not consider geographical factors that can play a significant role in sexual health outcomes, thus missing opportunities to effectively address localized epidemics or identify social and geographical determinants of health. For example, in the United States, geographic considerations can unearth the ongoing consequences of historical injustices such as race/ethnicity-related violence, regional bias, and mistrust. These factors contribute to health disparities and highlight the need for a more inclusive approach that considers geography in order to achieve health equity. Additionally, because the 5 and 6 Ps primarily record sexual behavior, they may also increase the likelihood that vulnerabilities to HIV are framed solely as individual risk, reinforcing stigma and shame and bypassing opportunities to address structural factors. Risk-based approaches to sexual health discussions can ultimately undermine trust and limit opportunities for affirming, equity-centered care [16–18].

To bridge the gaps identified in traditional models and provide deeper insights into the intricate nature of sexual health, we propose an 8 Ps framework that expands on the granular recording of behaviors by incorporating two additional Ps: Proximity and Perspective. This new framework permits exploration of contexts, beliefs, perceptions, and reason-based decisions that underpin sexual health outcomes, drive sexual health choices, and amplify vulnerabilities. Our expanded model provides healthcare providers with a comprehensive tool that allows a more holistic approach to patient care.

## 2 | The 8 Ps Framework: An Overview

**FIGURE 1 |:**
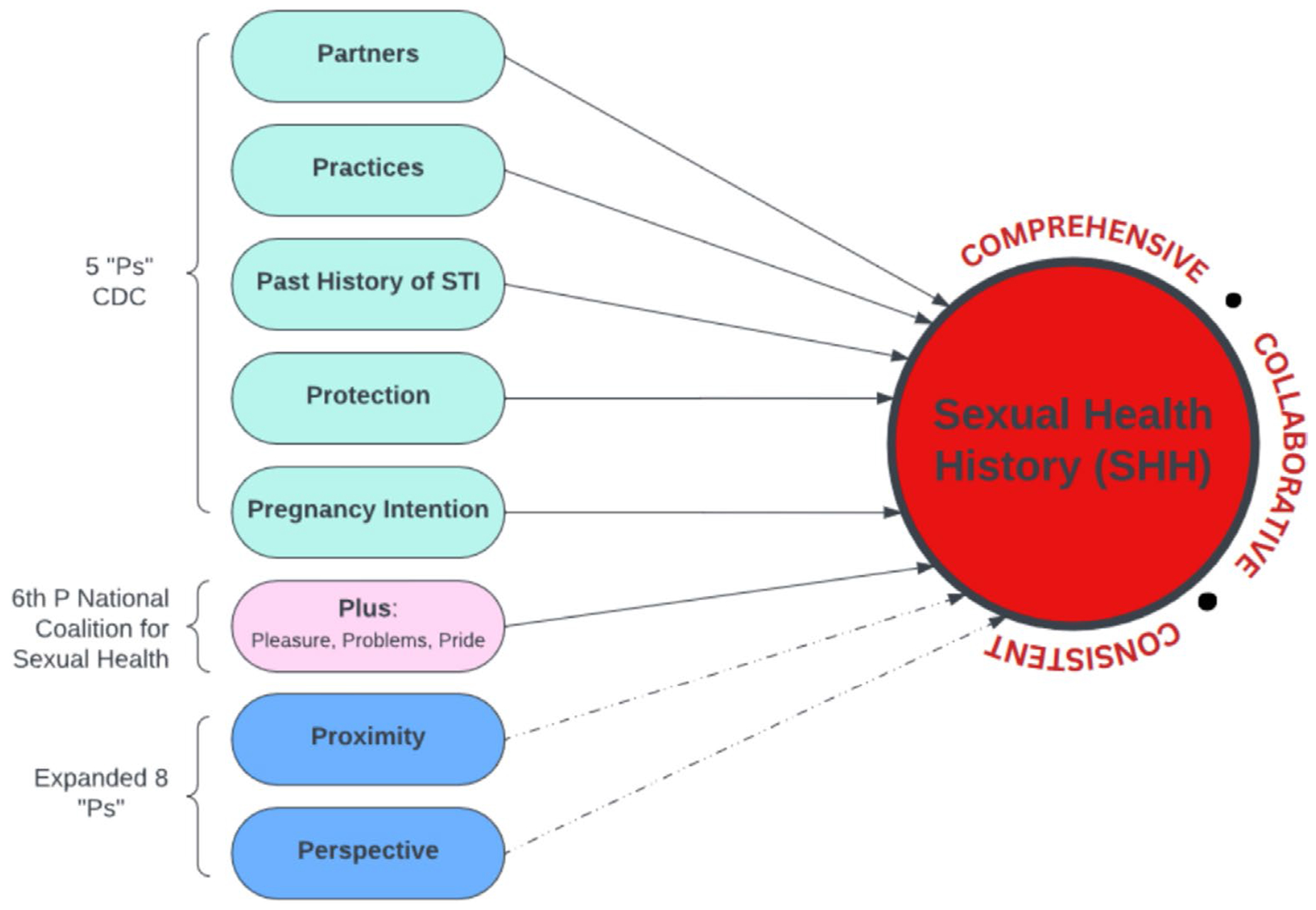
8 Ps framework.

The 8 Ps framework introduces two new elements to patient-provider sexual health conversations. Herein, we provide an overview of the current 6 P model (Partners, Practices, Protection from STIs, Past history of STIs, Pregnancy plans, Plus) and introduce the rationale for expansion. In this section, we have intentionally removed the language of risk, replacing it with that of vulnerability or susceptibility (Figure 1).

The development of the additional *P’s*—*Proximity* and *Perspectives*—emerged from over 15 years of clinical practice, public health scholarship, and community-engaged research focused on HIV prevention and sexual health promotion in Black communities. Through this work, it became clear that behavior-centered frameworks, while useful, insufficiently capture the structural, environmental, and cultural contexts that influence sexual health outcomes and patient–provider communication.

*Proximity* reflects evidence that geography, neighborhood resources, and sexual networks strongly shape vulnerability to HIV and other STIs. Social epidemiology and place-based health research consistently demonstrate that environmental context—including healthcare access, housing stability, and community-level prevalence—plays a pivotal role in outcomes [19–23].

*Perspectives* recognizes that individual beliefs, values, and lived experiences shape sexual decision-making and communication with providers. Drawing from qualitative studies, community-based interventions, and patient narratives, we identified the importance of integrating belief systems and cultural frameworks into sexual health discussions to foster trust and reduce stigma [24–27].

Thus, these additions are not hypothetical but grounded in both published evidence and extensive clinical and research experience. By broadening the framework, the 8 Ps aim to strengthen communication, reduce stigma, and align SHHs with contemporary understandings of social determinants of health.

### 2.1 | Partners

“Partners” comprises an individual’s unique sexual partners over time, including their sex/gender, as well as the number and nature of relationships (e.g., monogamous, polyamorous, casual) with them. Understanding the dynamics of an individual’s partnerships allows for assessing an individual’s degree of potential sexual health vulnerabilities and making recommendations for prevention strategies/recommendations. “Partners” also include direct questions about an individual’s partner’s HIV status. Such questions encourage thoughtful reflection and conversation about the patient’s knowledge and awareness of their partner’s status as well as their confidence in asking their partner questions, setting the stage for a conversation about PrEP or PEP [28–31].

### 2.2 | Practices

“Practices” encompass the range of sexual activities in which an individual engages, such as vaginal, oral, and/or anal intercourse, along with other activities that may increase susceptibility to STIs/HIV, including partner sharing, sex work, and participation in sexual fantasy events. These situations may heighten susceptibility to STIs/HIV due to increased exposure through overlapping sexual networks (i.e., concurrency and inconsistent condom use), higher STI prevalence and structural vulnerabilities among sex workers, and factors such as substance use and coercion that undermine safe practices and potentially accelerates transmission [32–36]. These activities, while potentially pleasurable, also require proper education and prevention strategies to ensure safety. Therefore, this information is crucial for healthcare providers to tailor prevention strategies effectively and ensure individual comfort and consent during sexual activities.

### 2.3 | Past History of STIs

This category constitutes a comprehensive and thorough history of STI and HIV testing. Information about all prior testing, regardless of result, is indicative of past experiences that could affect current health status and allows insight into the patient’s previous engagement or adherence with recommended testing and treatment. This line of questioning can inform a discussion of reasons for testing and recommended frequency of testing based on the individual’s answers to the remaining P’s. Because some past infections can have lasting physiological and psychological effects (i.e., infertility, increased susceptibility to new infections), a complete past history assessment permits a more nuanced approach to recommending current healthcare strategies (e.g., by identifying individuals who may have specific reasons to choose biomedical prevention approaches, including PrEP and PEP).

### 2.4 | Protection

The “Protection from STIs” component focuses on the individual’s proactive choices and frequency of use of various preventive measures (e.g., condoms, dental dams, PrEP, vaccinations) to prevent STIs/HIV. This category allows the provider to assess the patient’s awareness and proactivity, thereby identifying opportunities for enhanced preventive education and practice adoption when necessary.

### 2.5 | Pregnancy Intention

“Pregnancy intention” refers to an individual’s current plans, goals, and attitudes toward becoming pregnant. Pregnancy intentions often dictate choice of contraception and can influence sexual practices. For example, an individual actively trying to conceive a pregnancy may forgo certain barrier methods, thus potentially altering their STI/HIV susceptibility. At the same time, there are safer conception and fertility attainment options such as PrEP, timed condomless intercourse during the ovulation period when HIV status is unknown or at detectable levels [37–39], antiretroviral therapy for HIV-positive partners to maintain viral suppression [37, 38], and assisted reproductive technologies like sperm washing combined with intrauterine insemination (IUI) or in vitro fertilization (IVF)—all of which have been shown to reduce HIV/STI risk while supporting reproductive goals [40]. This category is essential as it offers nuanced insights into a person’s reproductive health choices, desires, and sexual health needs. Understanding an individual’s pregnancy intentions enables healthcare providers to guide discussions about family planning, contraception, and fertility in a targeted manner.

### 2.6 | Plus

“Plus” refers to an individual’s sexual satisfaction and the overall quality of their sexual experiences. This component provides a more holistic view of sexual health, reaching beyond health burdens to include emotional and psychological aspects of sexual well-being. Satisfying sexual experiences can encourage consistent use of protection and promote a more positive, proactive approach to sexual health. In addition, conversations about sexual satisfaction can bring to light underlying issues that may impact overall sexual health, such as sexual dysfunction, psychological barriers (i.e., sexual distress), or relational challenges.

### 2.7 | Proximity

Understanding the geographical context of an individual’s sexual activities—termed “Proximity”—is crucial and plays a pivotal role in shaping sexual health outcomes, particularly in efforts to ending the HIV epidemic (EHE) [41] in the United States. EHE initiatives aim to substantially reduce HIV infections in the United States by 2030 in 57 priority jurisdictions, characterized by populations with high incidence of STIs and HIV, combined with low HIV viral suppression and HIV testing/screening. In addition to, and often as a byproduct, broader social and structural determinants of health in the United States are closely associated with geography. Geographical locations may intersect with increased poverty rates, disproportionately low income rates, lack of expanded Medicaid, and disparate policing leading to justice involvement for individuals and their romantic partners. These intersections are closely associated with increased rates of HIV [42]. The social and structural linkages to geography are well documented. The current SHH fails to consider “geobehavioral vulnerabilities to HIV,” a term coined by Brawner et al. [22]. Because proximity is often an indicator of choice of sexual partners [43, 44], where you live and have sex can increase sexual health vulnerabilities. Proximity also accounts for the potential effects of migration or frequent travel on sexual health [45, 46], including different exposure to sexual health vulnerabilities (e.g., travel to areas of high prevalence). An individual’s geography may also suggest a difference in access to sexual healthcare services [47] (e.g., condom availability and limited access to sexual health care in rural communities). Infrastructure shapes proximity by determining the availability and accessibility of sexual healthcare services, transportation networks, and community resources, which in turn influence how close or distant individuals are to essential services.

Understanding of an individual’s proximity to key priority areas and the spatial dimensions of sexual health outcomes guide targeted interventions, especially as sexual networks today extend across broader geographies by spanning cities, states, and countries where local epidemiological contexts and structural conditions may influence vulnerability within these expanded networks. A key aspect of sexual health education in considering geographical contributors to sexual health involves communicating potential increased vulnerabilities to STI and HIV transmission in areas with high infection rates and low viral suppression. This conversation also opens the door to discuss the concept of U=U, which is crucial for understanding and managing sexual health vulnerabilities related to HIV and mitigating stigma [7].

To aid healthcare providers and facilitate understanding of proximity in sexual health assessments, we offer targeted questions (Table 1). These questions are designed for patient–provider bidirectional communication and learning. By exploring proximity, healthcare providers and patients gain valuable insights into how geographical determinants of health impact sexual health, leading to more informed and effective prevention strategies.

### 2.8 | Perspective

“Perspective” in sexual health assessment refers to the intersection of personal, cultural, and social beliefs that influence an individual’s decisions and attitudes toward sexual health, including the individual’s and their provider’s perception of “risk.” In populations (i.e., Black women) disproportionately affected by HIV and other STIs, there often exists a disconnect or incongruence between perceptions of low or no risk and the reality of objective risks [29, 48, 49]. For instance, a person might feel secure in a monogamous relationship, not considering the possibility of their partner’s undisclosed sexual activities or known baseline HIV status. Similarly, providers may stereotype individuals by their profession, socioeconomic status, or behaviors/attributes, leading to bias [50–52]. These biases can influence the care and advice provided (i.e., gatekeeping who gets information about PrEP and testing). It is crucial, therefore, that providers (a) reflect on and nurture self-awareness of their biases regarding sexual and reproductive health, (b) take concrete efforts to mitigate their biases, and (c) assess each patient’s perspective of their personal vulnerabilities to conduct a gap analysis. By modeling nonjudgmental, bias-aware care, providers can influence how patients perceive their own risk, which helps patients reframe misconceptions, reduce internalized stigma, and engage more accurately with prevention strategies [53–55]. This gap analysis is a critical step in being able to tailor prevention messaging that resonates with the patient’s worldview while addressing their actual susceptibility. A thorough assessment involves providing information that fosters empathetic dialogue that respects and incorporates the patient’s beliefs and cultural influences. For example, a patient might be questioned about their perception of (a) their vulnerability to STIs/HIV and the basis for this perception, (b) factors that might increase or decrease their vulnerability, or (c) what behaviors of their own or by their partners might constitute consent.

Culture is also an important determinant of HIV vulnerability, and cultural and social norms influence protective behaviors [56]. For example, religious beliefs may prohibit or discourage discussions related to sexuality and condom use, promoting norms that increase vulnerability to HIV and limit the capability of implementing self-protective behaviors [57]. Historical and contemporary medical abuses in Black communities have led to medical distrust and therefore cause concerns for Black women considering biomedical prevention options such as PrEP [58]. Societal norms may also affect whether healthcare providers collect sexual health information from patients they do not view as sexually vulnerable, such as married or older patients. These examples underscore the importance of the *Perspectives* component in our expanded SHH framework, as understanding an individual’s cultural, historical, and social context is essential for accurately assessing sexual health needs, tailoring prevention strategies, and fostering patient-provider trust [59].

We propose a set of insightful questions (Table 1) to help healthcare providers align care strategies with the patient’s worldview, address misconceptions, and foster a more trusting and collaborative relationship with patients. These questions are designed to unravel the layered beliefs and perceptions that influence an individual’s approach to sexual health. By exploring the answers to these questions, healthcare providers can gain a deeper understanding of their patients’ perspectives, enabling them to offer more personalized and impactful care.

## 3 | Conclusion

Traditional SHH frameworks may be inadequate for capturing the complex nature of human sexuality. Our expanded 8 Ps framework offers a comprehensive approach that extends beyond documenting behaviors by identifying the contexts, beliefs, perceptions, and geographical factors which influence sexual health. In addition to assessing types and number of partners, this framework allows healthcare providers to gain a nuanced understanding of the belief systems and cultural influences that shape patients’ perceptions of sexual health and risk, thus (a) bridging the gap between perceived and actual vulnerabilities; (b) enabling tailored, effective, personalized approaches to each patient’s care; and (c) offering a pathway to more consistent, targeted, and empathetic interventions, particularly for populations most susceptible to HIV and other STIs.

Although we focused primarily on SHHs as a means of prevention and early detection of STIs/HIV, the utility of the SHH extends further. Sex and sexual pleasure are an extension of overall health; therefore, healthcare providers should consider the SHH not solely as an assessment of an individual’s vulnerability to STIs/HIV but as a means to encourage a sex-positive mindset, promote sexual pleasure, and increase sexual health equity [60]. By embracing the expanded 8 Ps framework, we advocate for a transformative approach to sexual health—one that is holistic, empathetic, and equitable. This shift is vital in fostering environments where sexual health is viewed as a positive and integral component of human health, aligning with the principles of sexual rights and well-being [26]. Embracing this framework represents a significant stride toward enriching the landscape of sexual health care and education. Finally, a concurrent shift from risk-based language to a reasons-based approach [16, 61] is necessary to destigmatize and remove shame from sexual health conversations, care, and treatment.

## 3.1 | Call to Action

### 3.1.1 | Researchers

Efforts to validate and refine the 8 Ps framework are necessary. Research should focus on cross-examining this framework across diverse demographics, cultures, and geographies to offer valuable insights and refinement. Additionally, interdisciplinary research that incorporates the key stakeholders on the healthcare team and explores methodologies for efficiently collecting and documenting the SHH in patient records is vital. Addressing common barriers like time constraints and knowledge gaps may further streamline this process and ultimately improve patient outcomes.

### 3.1.2 | Healthcare Providers

We strongly urge healthcare providers to integrate the 8 Ps framework into their clinical practice. The depth of insight it offers can significantly enhance patient care, particularly for those most vulnerable to HIV and other STIs. To aid in this integration, we recommend development and participation in specialized training sessions or workshops that provide in-depth understanding and practical application of this framework. In areas where HIV prevalence is high and viral suppression is low, healthcare providers should encourage biomedical prevention of HIV with either daily or long-acting injectable PrEP. Healthcare providers can use the AIDSVU interactive map (https://map.aidsvu.org/map) to access and share county-specific data on prevalence, viral suppression, and PrEP use, enriching patient discussion and customizing care plans, especially when acknowledging partners from different areas (i.e., counties, states, countries, etc.).

### 3.1.3 | Academic Institutions

Healthcare training programs (e.g., medicine, nursing, pharmacy) could consider incorporating the 8 Ps framework into their curricula after sufficiently testing and validating. This integration can be achieved through interactive case studies, real-world scenario simulations, and comprehensive modules that emphasize patient-provider communication. Such an approach helps to ensure that the next generation of healthcare providers is equipped to address the complexities of modern sexual health.

### 3.1.4 | Policy

Healthcare leaders and policymakers should consider how the 8 Ps framework can influence healthcare policy, specifically for health insurance carriers. There is an opportunity to translate research findings advocating for the integration of the collection and comprehensive documentation of SHH and sexual health education into reimbursement models. Such policy changes could incentivize thorough and empathetic patient care, potentially leading to better health outcomes.

## Figures and Tables

**TABLE 1 |:** 8 Ps framework to take a sexual health history.

Proximity	Providers should know baseline prevalence and viral suppression data from the community they serve. • Where do your sexual partners live? (i.e., zip code and/or county) • Do you travel to meet sex partner(s)? How often? • Are you aware of the HIV/STI prevalence rates in your area or areas you travel to for partners? • How does your location affect your access to sexual health services? • Have you experienced any changes in your sexual practices due to your location or travel?
Perspectives	• What do you think your vulnerability to STIs/HIV is, and why do you feel that way? • What would make you feel less vulnerable to STIs/HIV? • Can you describe your idea of consent in sexual relationships? • How do your cultural or social beliefs impact your decisions about sexual health? • Are there any personal or cultural factors that influence how you approach STI/HIV prevention?
Partners	• Could you tell me about your current relationship(s)? • Are you currently having sex, like in the last month—6 months? This could be oral, anal, or vaginal. • Have you ever had sex of any kind with someone? • What is the gender of your partners? • How many sex partners have you had in the past 6 months? • Do you or your sex partners have other sex partners? • Do you know your partner’s HIV status?
Practices	• What kinds of sexual contact do you have or have had in the past? (vaginal, oral, anal [insertive or receptive]) • Have you or your partners used drugs? • Have you or your partners been employed in sex work?
Protection from STIs	• What has your partner told you about their history of STIs? • How often do you and your partner discuss STI prevention? • What prevention tools do you use? (condoms, dental dams, etc.) • How often do you use prevention, which one, and what made you choose this method? • Are you aware of PrEP and PEP? If so, have you ever used it or considered using it?
Past history of STIs	• Have you ever been tested for STIs and HIV? When was the last time? Would you like to be tested? • Have you ever been diagnosed with an STI? When? Did you complete treatment? Did you discuss with your partner? Did they get treated? • Has your current partner or former partners been diagnosed and treated for an STI?
Pregnancy intention	• Do you and your partner want to have (more) children at some point? • When do you think that might be? • How important is it to prevent pregnancy? • Are you and your partner using contraception, if so, what kind?
Plus	
Pleasure	• How is your sex life going?
Problems	• Are you or your partners having any problems with sexual functioning? (e.g., pain, low sex drive, vaginal dryness, lack of erection, lack of orgasm)
Pride	• What support, if any, do you have (need) from your family and friends about your gender identity or sexual orientation? How can I support you?

## References

[1] HHS, “Sexually Transmitted Infections (STIs),” accessed September 9, 2025, https://www.hhs.gov/programs/topic-sites/sexually-transmitted-infections/index.html.

[2] CDC, “Eliminating HIV as a Global Public Health Threat,” Vital-Signs, CDC, accessed September 9, 2025, https://www.cdc.gov/vitalsigns/global-hiv/index.html.

[3] CDC, “Fast Facts: HIV in the US by Race and Ethnicity,” HIV, CDC, accessed September 9, 2025, https://www.cdc.gov/hiv/data-research/facts-stats/race-ethnicity.html.

[4] Palaiodimos L, Herman HS, Wood E, , “Practices and Barriers in Sexual History Taking: A Cross-Sectional Study in a Public Adult Primary Care Clinic,” Journal of Sexual Medicine 17, no. 8 (2020): 1509–1519, 10.1016/j.jsxm.2020.05.004. 32605821

[5] Ford N, Mayer KH, and World Health Organization Postexposure Prophylaxis Guideline Development Group, “World Health Organization Guidelines on Postexposure Prophylaxis for HIV: Recommendations for a Public Health Approach,” Clinical Infectious Diseases 60, no. 3 (2015): S161–S164, 10.1093/cid/civ068. 25972497

[6] Bunting SR, Hunt B, Boshara A, , “Examining the Correlation Between PrEP Use and Black:White Disparities in HIV Incidence in the Ending the HIV Epidemic Priority Jurisdictions,” Journal of General Internal Medicine 38, no. 2 (2023): 382–389, 10.1007/s11606-022-07687-y. 35678988 PMC9905374

[7] Bor J, Fischer C, Modi M, , “Changing Knowledge and Attitudes Towards HIV Treatment-As-Prevention and “Undetectable = Untransmittable”: A Systematic Review,” AIDS and Behavior 25, no. 12 (2021): 4209–4224, 10.1007/s10461-021-03296-8. 34036459 PMC8147591

[8] Okoli C, Van de Velde N, Richman B, , “Undetectable Equals Untransmittable (U = U): Awareness and Associations With Health Outcomes Among People Living With HIV in 25 Countries,” Sexually Transmitted Infections 97, no. 1 (2021): 18–26, 10.1136/sextrans-2020-054551. 32732335 PMC7841488

[9] National Institutes of Health (NIH), “The Science is Clear: With HIV, Undetectable Equals Untransmittable,” accessed September 10, 2025, https://www.nih.gov/news-events/news-releases/science-clear-hiv-undetectable-equals-untransmittable.

[10] National Institute of Allergy and Infectious Diseases (NIAID), “HIV Undetectable=Untransmittable (U=U), or Treatment as Prevention,” May 21, 2019, https://www.niaid.nih.gov/diseases-conditions/treatment-prevention.

[11] CDC, “Discussing Sexual Health With Your Patients,” HIV Nexus, CDC, accessed September 9, 2025, https://www.cdc.gov/hivnexus/hcp/sexual-history/index.html.

[12] National Coalition for Sexual Health, “Sexual Health Questions to Ask All Patients,” accessed September 9, 2025, https://www.nationalcoalitionforsexualhealth.org/tools/for-healthcare-providers/sexual-health-questions-to-ask-all-patients/.

[13] Gautam R and Orrino J, “Improving Chlamydia Risk Screening by Using the CDC’S 5 Ps Approach to Sexual Health History,” Journal of the American Association of Nurse Practitioners 35, no. 7 (2023): 441–448, 10.1097/JXX.0000000000000829. 36728254

[14] Golub SA, Gamarel KE, and Lelutiu-Weinberger C, “The Importance of Sexual History Taking for PrEP Comprehension Among Young People of Color,” AIDS and Behavior 21, no. 5 (2017): 1315–1324, 10.1007/s10461-016-1512-9. 27475944 PMC5280583

[15] Savoy M, O’Gurek D, and Brown-James A, “Sexual Health History: Techniques and Tips,” American Family Physician 101, no. 5 (2020): 286–293. 32109033

[16] Johnson R, Duroseau B, Randolph S, and Chandler R, “Reasons Over Risks: NPs and HIV Prevention for Black Women,” Journal for Nurse Practitioners 20, no. 3 (2024): 104931, 10.1016/j.nurpra.2024.104931.

[17] Positively Aware, “Say Goodbye to ‘Risk’,” accessed September 9, 2025, https://www.positivelyaware.com/articles/say-goodbye-‘risk’.

[18] Marcus JL and Snowden JM, “Words Matter: Putting an End to “Unsafe” and “Risky” Sex,” Sexually Transmitted Diseases 47, no. 1 (2020): 1–3, 10.1097/OLQ.0000000000001065. 31517770 PMC6953392

[19] Adimora AA and Schoenbach VJ, “Social Context, Sexual Networks, and Racial Disparities in Rates of Sexually Transmitted Infections,” Journal of Infectious Diseases 191, no. 1 (2005): S115–S122, 10.1086/425280. 15627221

[20] Adimora AA and Schoenbach VJ, “Social Determinants of Sexual Networks, Partnership Formation, and Sexually Transmitted Infections,” in The New Public Health and STD/HIV Prevention, ed. Aral SO, Fenton KA, and Lipshutz JA (Springer New York, 2013), 13–31, 10.1007/978-1-4614-4526-5_2.

[21] Newmyer L, Evans M, and Graif C, “Socially Connected Neighborhoods and the Spread of Sexually Transmitted Infections,” Demography 59, no. 4 (2022): 1299–1323, 10.1215/00703370-10054898. 35838157 PMC9707946

[22] Brawner BM, Kerr J, Castle BF, , “A Systematic Review of Neighborhood-Level Influences on HIV Vulnerability,” AIDS and Behavior 26, no. 3 (2022): 874–934, 10.1007/s10461-021-03448-w. 34480256 PMC8415438

[23] Logan JJ, Jolly AM, and Blanford JI, “The Sociospatial Network: Risk and the Role of Place in the Transmission of Infectious Diseases,” PLoS One 11, no. 2 (2016): e0146915, 10.1371/journal.pone.0146915. 26840891 PMC4739620

[24] Mohd NF. Tohit and Haque M, “Forbidden Conversations: A Comprehensive Exploration of Taboos in Sexual and Reproductive Health,” Cureus 16 (2024): e66723, 10.7759/cureus.66723. 39139803 PMC11319820

[25] Arousell J and Carlbom A, “Culture and Religious Beliefs in Relation to Reproductive Health,” Best Practice & Research. Clinical Obstetrics & Gynaecology 32 (2016): 77–87, 10.1016/j.bpobgyn.2015.08.011. 26542927

[26] Stewart JM, Sommers MS, and Brawner BM, “The Black Church, Sexual Health, and Sexuality: A Conceptual Framework to Promote Health Through Faith-Based Organizations,” Family & Community Health 36, no. 3 (2013): 269–279, 10.1097/FCH.0b013e318292eb2d. 23718962

[27] Fortenberry JD, “Trust, Sexual Trust, and Sexual Health: An Interrogative Review,” Journal of Sex Research 56, no. 4–5 (2019): 425–439, 10.1080/00224499.2018.1523999. 30289286

[28] Carter G, Staten IC, Woodward B, Mahnke B, and Campbell J, “PrEP Prescription Among MSM U.S. Military Service Members: Race and Sexual Identification Matter,” American Journal of Men’s Health 16, no. 6 (2022): 15579883221133891, 10.1177/15579883221133891. PMC9630896 36317720

[29] Chandler R, Guillaume D, Wells J, and Hernandez N, “Let Me Prep You to PREP Me: Amplifying the Voices of Black Women and Their Providers to Consider PrEP as an HIV Prevention Option,” International Journal of Environmental Research and Public Health 19, no. 3 (2022): 1414, 10.3390/ijerph19031414. 35162438 PMC8835000

[30] Young LE, Baird A, and Schneider JA, “Diagnosing PrEP Communication Self-Efficacy in a Community-Based Peer Leader Intervention for Black Sexual Minority Men,” AIDS and Behavior 26, no. 11 (2022): 3747–3760, 10.1007/s10461-022-03704-7. 35583572 PMC9550693

[31] Irie W, Mahone A, Nakka R, and Ghebremichael M, “Confidence in Ability to Communicate With Sexual Partners About PrEP Among Black Cisgender Women,” AIDS Education and Prevention 35, no. 5 (2023): 333–346, 10.1521/aeap.2023.35.5.333. 37843905

[32] Gorbach PM, “Transmission of STIs/HIV at the Partnership Level: Beyond Individual-Level Analyses,” Journal of Urban Health 80 (2003): iii15–iii25, 10.1093/jurban/jtg079. 14713668 PMC3456259

[33] Mah TL and Shelton JD, “Concurrency Revisited: Increasing and Compelling Epidemiological Evidence,” Journal of the International AIDS Society 14, no. 1 (2011): 33, 10.1186/1758-2652-14-33. 21689437 PMC3133533

[34] Senn TE, Carey MP, Vanable PA, Coury-Doniger P, and Urban M, “Sexual Partner Concurrency Among STI Clinic Patients With a Steady Partner: Correlates and Associations With Condom Use,” Sexually Transmitted Infections 85, no. 5 (2009): 343–347, 10.1136/sti.2009.035758. 19204019 PMC2935200

[35] Rusakova M, Rakhmetova A, and Strathdee SA, “Why Are Sex Workers Who Use Substances at Risk for HIV?,” Lancet 385, no. 9964 (2015): 211–212, 10.1016/S0140-6736(14)61042-4. 25059944

[36] Argento E, Goldenberg S, and Shannon K, “Preventing Sexually Transmitted and Blood Borne Infections (STBBIs) Among Sex Workers: A Critical Review of the Evidence on Determinants and Interventions in High-Income Countries,” BMC Infectious Diseases 19, no. 1 (2019): 212, 10.1186/s12879-019-3694-z. 30832596 PMC6399876

[37] Saleem HT, Narasimhan M, Denison JA, and Kennedy CE, “Achieving Pregnancy Safely for HIV-Serodiscordant Couples: A Social Ecological Approach,” Journal of the International AIDS Society 20, no. S1 (2017): 21331, 10.7448/IAS.20.2.21331. 28361502 PMC5577721

[38] Sun L, Liu A, Li J, , “Is PrEP Necessary During Natural Conception in HIV-1-Serodiscordant Couples on ART With Suppressed Viral Load? A Retrospective Cohort Analysis,” BMC Infectious Diseases 20, no. 1 (2020): 195, 10.1186/s12879-020-4912-4. 32138673 PMC7059657

[39] Brooks JT, Kawwass JF, Smith DK, , “Effects of Antiretroviral Therapy to Prevent HIV Transmission to Women in Couples Attempting Conception When the Man Has HIV Infection—United States, 2017,” MMWR. Morbidity and Mortality Weekly Report 66, no. 32 (2017): 859–860, 10.15585/mmwr.mm6632e1. 28817552 PMC5657665

[40] Matthews LT, Beyeza-Kashesya J, Cooke I, , “Consensus Statement: Supporting Safer Conception and Pregnancy for Men and Women Living With and Affected by HIV,” AIDS and Behavior 22, no. 6 (2018): 1713–1724, 10.1007/s10461-017-1777-7. 28501964 PMC5683943

[41] HIV, “EHE Overview,” accessed September 10, 2025, https://www.hiv.gov/federal-response/ending-the-hiv-epidemic/overview.

[42] Nwangwu-Ike N, Jin C, Gant Z, Johnson S, and Balaji AB, “An Examination of Geographic Differences in Social Determinants of Health Among Women With Diagnosed HIV in the United States and Puerto Rico, 2017,” Open AIDS Journal 15, no. 1 (2021): 10–20, 10.2174/1874613602115010010.

[43] Brawner BM, Guthrie B, Stevens R, Taylor L, Eberhart M, and Schensul JJ, “Place Still Matters: Racial/Ethnic and Geographic Disparities in HIV Transmission and Disease Burden,” Journal of Urban Health 94, no. 5 (2017): 716–729, 10.1007/s11524-017-0198-2. 28879489 PMC5610132

[44] Nunn A, Yolken A, Cutler B, , “Geography Should Not Be Destiny: Focusing HIV/AIDS Implementation Research and Programs on Microepidemics in US Neighborhoods,” American Journal of Public Health 104, no. 5 (2014): 775–780, 10.2105/AJPH.2013.301864. 24716570 PMC3987607

[45] Cassels S, Cerezo A, Reid SC, Rivera DB, Loustalot C, and Meltzer D, “Geographic Mobility and Its Impact on Sexual Health and Ongoing HIV Transmission Among Migrant Latinx Men Who Have Sex With Men,” Social Science & Medicine 320 (2023): 115635, 10.1016/j.socscimed.2022.115635. 36640703 PMC10866558

[46] Davies M, Lewis NM, and Moon G, “Sexuality, Space, Gender, and Health: Renewing Geographical Approaches to Well-Being in Lesbian, Gay, Bisexual, Transgender, and Queer Populations,” Geography Compass 12, no. 5 (2018): e12369, 10.1111/gec3.12369.

[47] Shacham E, Nelson EJ, Schulte L, Bloomfield M, and Murphy R, “Condom Deserts: Geographical Disparities in Condom Availability and Their Relationship With Rates of Sexually Transmitted Infections,” Sexually Transmitted Infections 92, no. 3 (2016): 194–199, 10.1136/sextrans-2015-052144. 26567330

[48] Moore MP, Javier SJ, Maxwell ML, Dunn CE, and Belgrave FZ, ““You Put Yourself at Risk to Keep the Relationship:” African American Women’s Perspectives on Womanhood, Relationships, Sex and HIV,” Culture, Health & Sexuality 23 (2020): 1–16, 10.1080/13691058.2020.1815240. 32964793

[49] Khawcharoenporn T, Mongkolkaewsub S, Naijitra C, Khonphiern W, Apisarnthanarak A, and Phanuphak N, “HIV Risk, Risk Perception and Uptake of HIV Testing and Counseling Among Youth Men Who Have Sex With Men Attending a Gay Sauna,” AIDS Research and Therapy 16, no. 1 (2019): 13, 10.1186/s12981-019-0229-z. 31189481 PMC6560849

[50] Pleuhs B, Quinn KG, Walsh JL, Petroll AE, and John SA, “Health Care Provider Barriers to HIV Pre-Exposure Prophylaxis in the United States: A Systematic Review,” AIDS Patient Care and STDs 34, no. 3 (2020): 111–123, 10.1089/apc.2019.0189. 32109141 PMC7087402

[51] Mendez AD, Neelamegam M, and Griner SB, “Health Care Provider Discussions Regarding HIV/Sexually Transmitted Infection Risk Factors and Associations With HIV/Sexually Transmitted Infection Screening Among Men,” Archives of Sexual Behavior 52, no. 5 (2023): 2111–2121, 10.1007/s10508-023-02629-z. 37296333

[52] Fisher CB, Fried AL, Macapagal K, and Mustanski B, “Patient-Provider Communication Barriers and Facilitators to HIV and STI Preventive Services for Adolescent MSM,” AIDS and Behavior 22, no. 10 (2018): 3417–3428, 10.1007/s10461-018-2081-x. 29546468 PMC6139078

[53] Hull SJ, Tessema H, Thuku J, and Scott RK, “Providers PrEP: Identifying Primary Health Care Providers’ Biases as Barriers to Provision of Equitable PrEP Services,” Journal of Acquired Immune Deficiency Syndromes 88, no. 2 (2021): 165–172, 10.1097/QAI.0000000000002750. 34506359 PMC8577287

[54] Drumhiller K, Geter A, Elmore K, Gaul Z, and Sutton MY, “Perceptions of Patient HIV Risk by Primary Care Providers in High-HIV Prevalence Areas in the Southern United States,” AIDS Patient Care and STDs 34, no. 3 (2020): 102–110, 10.1089/apc.2019.0219. 32202928

[55] Geter A, Herron AR, and Sutton MY, “HIV-Related Stigma by Healthcare Providers in the United States: A Systematic Review,” AIDS Patient Care and STDs 32, no. 10 (2018): 418–424, 10.1089/apc.2018.0114. 30277814 PMC6410696

[56] Vitsupakorn S, Pierce N, and Ritchwood TD, “Cultural Interventions Addressing Disparities in the HIV Prevention and Treatment Cascade Among Black/African Americans: A Scoping Review,” BMC Public Health 23 (2023): 1748, 10.1186/s12889-023-16658-9. 37679765 PMC10485990

[57] El-Bassel N, Caldeira NA, Ruglass LM, and Gilbert L, “Addressing the Unique Needs of African American Women in HIV Prevention,” American Journal of Public Health 99, no. 6 (2009): 996–1001, 10.2105/AJPH.2008.140541. 19372518 PMC2679773

[58] Willie TC, Knight D, Baral SD, , “Where’s the “Everyday Black Woman”? An Intersectional Qualitative Analysis of Black Women’s Decision-Making Regarding HIV Pre-Exposure Prophylaxis (PrEP) in Mississippi,” BMC Public Health 22, no. 1 (2022): 1604, 10.1186/s12889-022-13999-9. 35999528 PMC9396836

[59] Lanier Y, Rivera-Cash D, Lavarin C, , “Application of the Unified Theory of Behavior to Strengthen Sexual Health Discussions Between Providers and Young Patients in the United States,” Perspectives on Sexual and Reproductive Health 56, no. 1 (2024): 4–15, 10.1111/psrh.12253. 38459825

[60] Bond KT and Radix AE, “Sexual Health and Well-Being: A Framework to Guide Care,” Medical Clinics of North America 108, no. 2 (2024): 241–255, 10.1016/j.mcna.2023.10.001. 38331477 PMC12877561

[61] ViiV Healthcare, “Risk to Reasons,” accessed September 9, 2025, https://viivhealthcare.com/en-us/supporting-the-hiv-community/positive-action/risk-to-reasons/.

